# Use of a novel technique to assess impact of age-related denervation on mouse soleus muscle function

**DOI:** 10.1007/s10522-023-10021-6

**Published:** 2023-02-15

**Authors:** Navneet N. Lal, Jon Cornwall, Philip W. Sheard

**Affiliations:** 1 Department of Physiology, School of Biomedical Sciences, University of Otago, Dunedin, New Zealand; 2 Centre for Early Learning in Medicine, Otago Medical School, University of Otago, Dunedin, New Zealand

**Keywords:** Skeletal muscle, Sarcopenia, Ageing, Denervation

## Abstract

Denervation contributes to loss of force-generating capacity in aged skeletal muscles, but problems with quantification of denervated fibers mean the precise impact of denervation on muscle function remains unclear. This study therefore looked to develop a reliable assay for identifying denervated muscle fibers, and used this to explore the impact of denervation on age-related force-generation in mouse skeletal muscle. Thirteen young (6-month-old) and 10 old (24-months-old) C57Bl/6 J female mice were utilized. Anaesthetized mice were infused with the fluorescent deoxyglucose analog 2[N-(7-nitrobenz-2-oxa-1,2-diaxol-4-yl)amino]-2-deoxyglucose (2-NBDG) and the tibial nerve was repeatedly stimulated to label active skeletal muscle fibers by activity-dependent uptake of 2-NBDG. Data on muscle force generation were acquired as part of the stimulation routine. Labeled muscles were removed, snap frozen, sectioned, and slide mounted. Sections were imaged to show accumulation of 2-NBDG in activated fibers and lack of 2-NBDG accumulation in quiescent (denervated) fibers, then processed using immunohistochemistry to allow collection of data on fiber number and morphology. Soleus muscles from older mice had nine times as many denervated fibers as those from young mice (average n = 36 vs 4, old vs young). Older muscles developed significantly more passive force and less specific force, but denervation only partly accounted for age-related deficits in specific force. Further investigations are required to definitively identify contributors to the decrease in force generation that remain unaccounted for.

## Introduction

Age-related skeletal muscle atrophy is accompanied by declines in muscle mass and function which threaten mobility and independence. The muscles of older individuals have been characterized as smaller (Larsson et al. 2018), stiffer (Wolfarth et al. 1997), weaker (Ballak et al. 2014a; Blough and Linderman 2000; Degens and Alway 2003; Kadhiresan et al. 1996; Linderman and Blough 2002; Rice et al. 2005), slower to develop force (Ballak et al. 2014a; Chan and Head 2010) and increasingly susceptible to injury (Pichavant and Pavlath 2014; Ramaswamy et al. 2011). Correlation of muscle strength with muscle mass reveals a relatively weak relationship (Blough and Linderman 2000; Kadhiresan et al. 1996) suggesting that loss of muscle mass (atrophy) is not the sole contributor to age-related changes in the force generating capacity of muscles. While many different elements contribute to declining force generation with increasing age, there is no framework that accounts precisely for all the loss that occurs.

Several non-atrophy related factors also contribute to age-related muscular weakness. These include muscle fiber denervation, reduced specific force (force per unit cross-sectional area), losses of force transmission, myosin isoform transformation, and changes in muscle structure (Larsson et al. 2018). Muscle fiber denervation is a prominent feature of muscle aging (Piasecki et al. 2018; Purves-Smith et al. 2012; Rowan et al. 2011, 2012; Sonjak et al. 2019), although denervated fibers may also exist in relatively young muscles (Urbanchek et al. 2001). Denervated muscle fibers contribute to muscle mass but since they are not recruited during nerve stimulation they do not contribute to force production, at least partially accounting for the poor correlation between muscle mass and force production.

Denervated fibers impact muscle function by presenting an inactive (compliant) path for the delivery of force from neighboring fibers (Sheard 2000). They also contribute to muscle mass and cross-sectional area, leading to underestimation of specific force in the muscles of older individuals. Adjusting area measurements for denervated fiber prevalence would assist calculations, however identification and quantification of denervated fibers is difficult. As a result, the exact contribution of denervation to force generating capacity in muscle aging is unknown.

Whilst several studies have documented fiber denervation (Chai et al. 2011; Piasecki et al. 2018; Purves-Smith et al. 2012; Rowan et al. 2011, 2012; Sonjak et al 2019; Valdez et al. 2010), and others have documented functional changes with age (Ballak et al. 2014a; Hill et al. 2020; Ham et al. 2020; Phillips et al. 1991), only one study has directly investigated both denervation and function in the same muscle and provided specific force estimates adjusted for denervated fiber prevalence. In this study (Urbanchek et al. 2001) putative denervated fibers were identified by immunohistochemical detection of NCAM, however two more recent studies have indicated that immunodetectable levels of NCAM do not provide a reliable indication of fiber denervation in rats or mice (Gillon and Sheard 2015; Hendrickse et al. 2018). The goals of this work were therefore to develop a new assay to more accurately quantify muscle fiber denervation, and to use this assay to further expand our understanding of the impact of age-related denervation on whole muscle function.

## Methods

### Animals

Soleus muscles were harvested from 13 young (6-month-old) and 10 old (24-months-old) C57Bl/6 J female mice, equivalent to 18 and 72 years of human ageing (Ballak et al 2014b). We used this strain because it shows age-related neuromuscular changes that are similar to those of humans in both degree and relative age of onset (Ballak et al. 2014b), and we used female mice because females commonly reach old age in our colony without age-related health problems. Mice were group housed in individually ventilated cages including plastic dome or cylindrical enrichment toys, food (standard mouse chow) and water which were available ad-libitum. All mice were kept under a 12-h light/dark cycle with lights on at 7 am. All experimental work was approved by Otago University Animal Ethics Committee.

### Surgical preparation

Prior to tissue collection, muscle force measurements were conducted on deeply anesthetized mice. The time of day and duration of all experiments were kept consistent. Anesthesia was achieved via an intraperitoneal injection of 30 mg.kg^−1^ sodium pentobarbitone. Once pedal withdrawal reflexes were abolished, the tail vein was cannulated with a 30-gauge needle and 300 µL of 300 µM 2-NBDG (2[N-(7-nitrobenz-2-oxa-1,2-diaxol-4-yl)amino]-2-deoxyglucose; Sapphire Bioscience, Australia) diluted in isotonic saline was infused. This procedure was to label active fibers with the fluorescent deoxyglucose analog 2-NBDG (see below).

Both legs were subsequently shaved and mice were placed in a prone position on a silicone elastomer (Sylgard 184, Dow Corning Corporation, MI, USA) molded platform. A longitudinal incision was made in the skin along the posterior midline of the left leg and retracted by fixation to the underlying Sylgard with 38 mm insect pins. The calcaneal tendon was carefully detached with surgical scissors and the triceps surae was gently reflected rostrally. The soleus was identified and its lateral margins were freed from the gastrocnemius via blunt dissection. Its distal tendon was entirely freed from the calcaneal tendon and attached to a straight tungsten wire in turn attached to an isometric force transducer (Harvard Bioscience MA, USA). The soleus nerve was identified and protected from stretching as the gastrocnemius was separated from the soleus. The portion of the calcaneal tendon attached to the gastrocnemius was pinned away to prevent interference with force measurements.

A bipolar stimulating electrode was positioned under the posterior tibial nerve and exposed soft tissues were covered in mineral oil to prevent desiccation. Stimulator (Grass SD9 Grass Instruments, RI, USA) settings were optimized to elicit the maximal force from a tetanic contraction. Force measurements were collected on Scope (v4.1.1) software via an ADInstruments PowerLab amplifier (AD Instruments, Dunedin, NZ) running on an Apple iMac computer (Apple Corp., CA, USA).

### Labeling denervated muscle fibers

Mice were given an intravenous bolus of 2-NBDG prior to stimulation, as outlined above. The presence of intravascular 2-NBDG was confirmed by popliteal artery fluorescence when excited under an epifluorescence-equipped Leica M3 (Leica Microsystems, Wetzlar, Germany) surgical microscope. 2-NBDG is a fluorescent glucose analogue which is taken up by active cells via the GLUT2 and GLUT4 transporters (Yamada et al. 2000) in response to activity induced by electrical stimulation (Toop et al. 1982). Unlike glucose, 2-NBDG is not metabolized once it has been taken up and accumulates in the cell according to the cell’s level of metabolic activity so active (stimulated) muscle fibers accumulate the fluorescent marker and denervated (inactive) fibers can be identified by their relative lack of 2-NBDG fluorescence. To drive 2-NBDG accumulation amongst innervated muscle fibers, the tibial nerve was continually stimulated to elicit a 500 ms tetanic contraction followed by 5 s of rest. The stimulation cycle was repeated continuously for 30 min in a manner similar to a previously published protocol (Toop et al. 1982) in which radioactive deoxyglucose was used to metabolically label stimulated fibers within a muscle.

### Functional measurements

On completion of the initial 2-NBDG loading regimen, functional measurements were made to permit characterization of the force produced at different nerve stimulation frequencies (to generate force-frequency curves) and of the active and passive tension at different muscle lengths (to generate length-tension curves) (Degens and Alway 2003). The stimulating electrode was initially placed on the belly and the muscle was stimulated directly, at different lengths to find the optimal length. The muscle was stimulated at supra maximal voltage to obtain peak muscle force as described previously (Ham et al. 2020; Schmidt et al. 2011). The electrode was then placed under the tibial nerve and force frequency measurements were obtained by stimulating with a train of 20 stimuli at a predetermined voltage and 10 ms pulse width. This was followed by a 30 s rest period. Recordings were repeated five times. The stimulus interval was gradually reduced from 500 to 12.5 ms (2-80 Hz) after every fifth recording. Length tension curves were generated by recording the force produced by the muscle at various lengths. The tibial nerve was stimulated at a predetermined frequency, voltage, and pulse width for a total of 250 ms. Stimulation voltages, frequencies and pulse widths were optimized to elicit the maximum force produced per contraction. Peak force production was taken from the muscle’s optimal length, which was determined retrospectively by finding the maxima of the length active-tension curve (Rassier et al. 1999).

### Tissue harvesting and euthanasia

Once force measurements were completed, the soleus was removed and pinned to a strip of Sylgard bathed in Ringer's lactate solution. The contralateral extensor digitorum longus was also harvested in a similar manner and served as a negative control for 2-NBDG-based visualization of denervated muscle fibers. Muscles were generously coated with Cryomatrix (Thermoscientific, MA, USA) and snap frozen in liquid nitrogen cooled L-isopentane. Mice were euthanized by sodium pentobarbitone overdose and cervical dislocation.

### Cryosectioning and immediate imaging

Cryomatrix-embedded left soleus muscles were first bisected at an oblique angle, then mounted upright to a specimen disk and sectioned at a plane selected to avoid tendons and to ensure inclusion of all fiber profiles (Lal et al. 2021). Sections were picked up onto poly-L-lysine coated microscope slides. Sections were kept in a light-tight slide holder whilst drying, and then imaged with a Spot-RT Slider CCD camera (Diagnostic Instruments, MI, USA) mounted to an Olympus BX-50 (Olympus Corp., Tokyo, Japan) upright compound microscope. Sections were illuminated with 470 nm wavelength light with a ρE-1 excitation system (CoolLED, Andover, UK) and viewed at 100 × magnification. The entire transverse sections of muscles were imaged by taking overlapping images which were later stitched together in Adobe Photoshop (Adobe Corp., CA, USA) as part of a photomerging routine. Tones and colors were not altered during this process. Once imaged, slides were stored at – 80 °C for immunostaining later.

### Immunofluorescent staining

Slides were defrosted at room temperature and then rehydrated in 50 mM tris buffered saline, which washed out the 2-NBDG. Slides were then incubated with rabbit polyclonal anti-Dystrophin at 1:200 dilution (ab15277, Abcam) overnight at 4 °C. The following morning, slides were washed in nine changes of Tris-buffered saline (TBS) (3 × in 50 mM TBS, 3 × in 100 mM TBS, 3 × in 50 mM TBS) and re-incubated with an AlexaFluor 568 conjugated goat anti-rabbit IgG secondary antibody (A-11034, 1:200 dilution; Life Technologies, CA, USA) for four hours at room temperature. Antibody concentrations and incubation times were optimized to work on fresh frozen mouse muscle tissue. Each staining batch also included a negative control in which the primary antibody was omitted from the immunodiluent. Once secondary antibody incubation was complete, slides were washed in another nine changes of TBS and cover-slipped using a 10:1 ratio of glycerol and TBS. The coverslip was sealed with clear nail polish. Sections were illuminated and photographed as described above.

### Image analysis

Digital micrographs were analyzed in a multistep manner that involved automated muscle fiber segmentation and manual refinement. Automated muscle fiber segmentation was done using a machine learning algorithm (Mask RCNN: recurrent convolutional neural network) (He et al. 2017) which was made publicly available by the Facebook Artificial Intelligence Research group (FAIR). This network was naïve to digital photomicrographs and required training. Training utilized transfer learning by loading network weights from when the creators initially trained it on the COCO dataset (Lin et al. 2014). Three hundred pre-annotated images were fed to the network and all layers were trained. The image and mask (annotations) dimensions were resized using the ‘pad64’ setting and augmented with flips, warps, skews, rotations, dropouts and noise (salt and pepper, gaussian blurring) using the ‘imgaug’ python library (Jung et al. 2020) to mitigate overfitting the dataset. The network was trained for 400 epochs with a learning rate of 0.001 and learning momentum of 0.9; similar settings were used to train the network for nuclear segmentation in previously published work (Johnson 2018). Training took seven full days on a custom-built machine with 32gb ram, intel core i7 8700 k CPU (3.70 GHz) and an NVIDIA GTX1080Ti GPU.

Once trained, digital photomicrographs of dystrophin or 2-NBDG fluorescence were fed through the network and segmented to yield binary masks for each muscle fiber. Masks were manually checked and refined where required, and used to measure fiber number and minimum feret diameter (cross-sectional area was inaccurate due to the oblique profile of fibers). ACSA was calculated from fiber number and cross-sectional area derived from minimum feret diameter (π[min. feret/2]^2^) in accordance with other publications (Gonzalez et al. 2000; Thomson and Gordon 2005). The manually classified masks were loaded into ImageJ and a comma separated values spreadsheet of numerical values was produced using the measure particles feature.

### Statistics

Active, passive, specific and denervation-adjusted specific force measurements were assessed for normality using D’Agostino & Pearson Omnibus normality test, and muscles from younger and older mice were subsequently compared using unpaired t-tests. Force-frequency and length-passive tension measurements were modelled using a 5-parameter logistic function and exponential growth curve fit using partial least squares method, respectively. The datasets were initially compared for ‘goodness of fit’ measured via the corrected Akaike information criterion (AICc), using either one or two (one each for younger and older groups) curves. Analyses of force frequency curves were conducted in GraphPad Prism (version 6.01).

## Results

### Whole muscle function

**Fig. 1:**
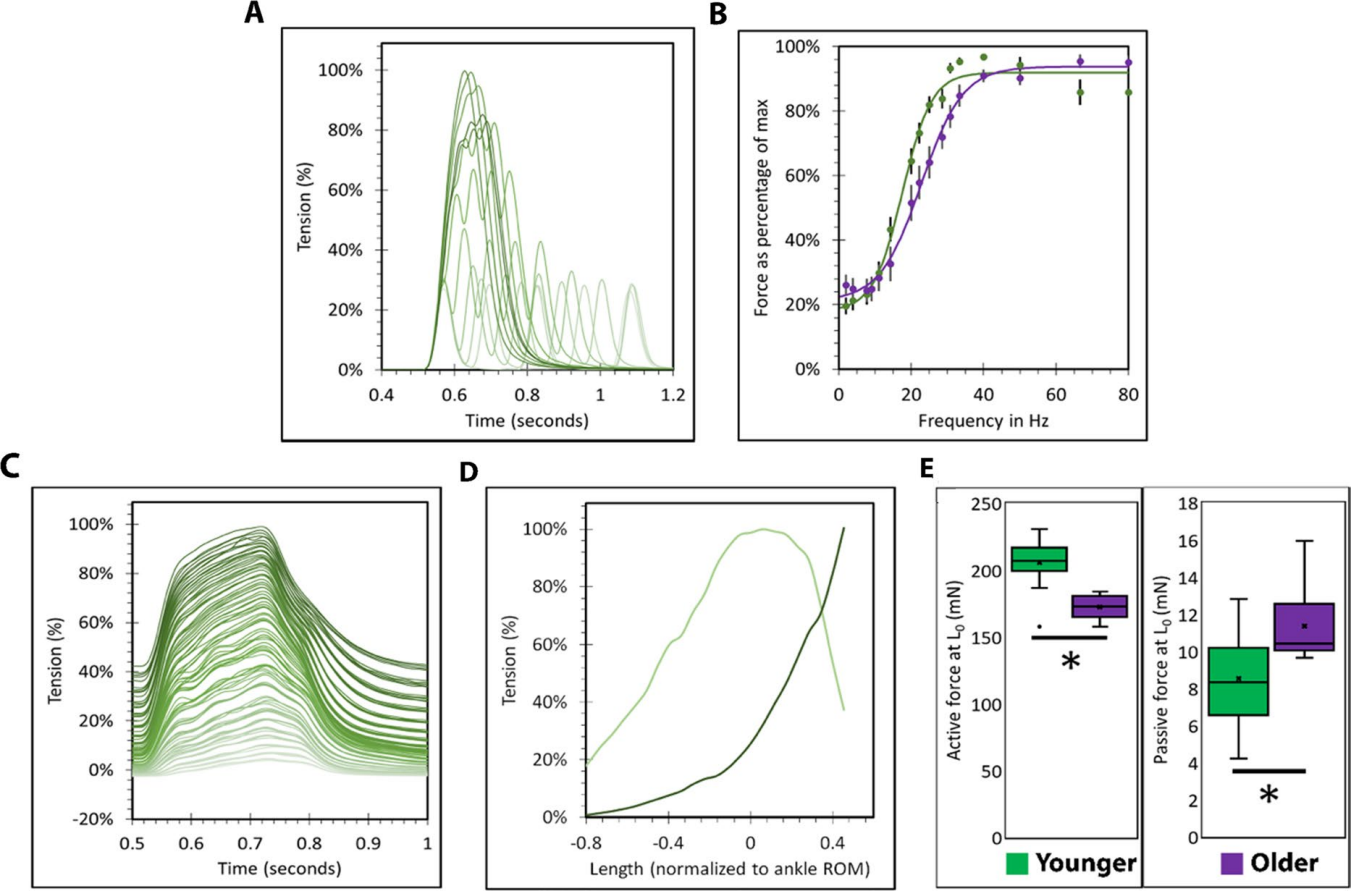
Force frequency curves, active and passive tension of soleus muscles from younger and older mice. The force frequency curves of muscles from younger and older mice were characterized by applying electrical stimulation at the tibial nerve at a range of frequencies. **A** Shows overlaid example traces. The force-frequency curves of muscles from older and younger mice were found to be different when modelled by a 5-parameter logistic function fit using least partial squares and analyzed for differences in the Akaike’s information criteria of one curve (for both younger and older) vs. two (one each for younger and older). **B** The slope center was rightward shifted and the gradient was shallower amongst soleus muscles from older mice (purple). The length tension curves of younger and older animals were also characterized by electrical stimulation at a range of muscle lengths (example traces overlaid in **C** and visualized in **D**). **E** At L_0_, the muscles of older mice produced less active and more passive force. N = 13 for younger and 10 for older mice. *p < 0.050

We measured four functional parameters across soleus muscles from younger and older mice: force-frequency (Fig. 1A-B), active tension and passive tension (Fig. 1C-D) and maximum tension developed at the muscle optimal length (L_0_) (Fig. 1E). Force frequency curves were fit to a 5-parameter (top, bottom, LogEC^50^, Hill slope and span) logistic function by least partial squares method. Akaike’s information criteria (AICc) were used to determine whether one (younger and older groups combined) or two curves (younger and older groups separated) modelled the data better. The difference in AICc between one and two curves was 45, indicating that two separate curves modelled the data better than one (Fig. 1). The force-frequency curve from the muscles of older mice differed from younger mice by two parameters (LogEC^50^ and Hill slope), indicating that it was right-shifted (frequency at half maximal force/LogEC^50^: 18[16–19] vs. 23[21–25]Hz; best fit value[95% confidence interval]) and shallower (Hill slope: 11[8–13] vs. 8[5–10]%.Hz^−1^; best fit value[95%CI]). The muscles of older mice produced less active (206 ± 17 vs. 172 ± 10mN at L_0_, p < 0.001, younger and older respectively) and more passive force (9 ± 3 vs. 11 ± 2mN at L_0_, p = 0.041, younger and older respectively) at L_0_ (Fig. 1E).

### Denervated muscle fiber quantification

**Fig. 2:**
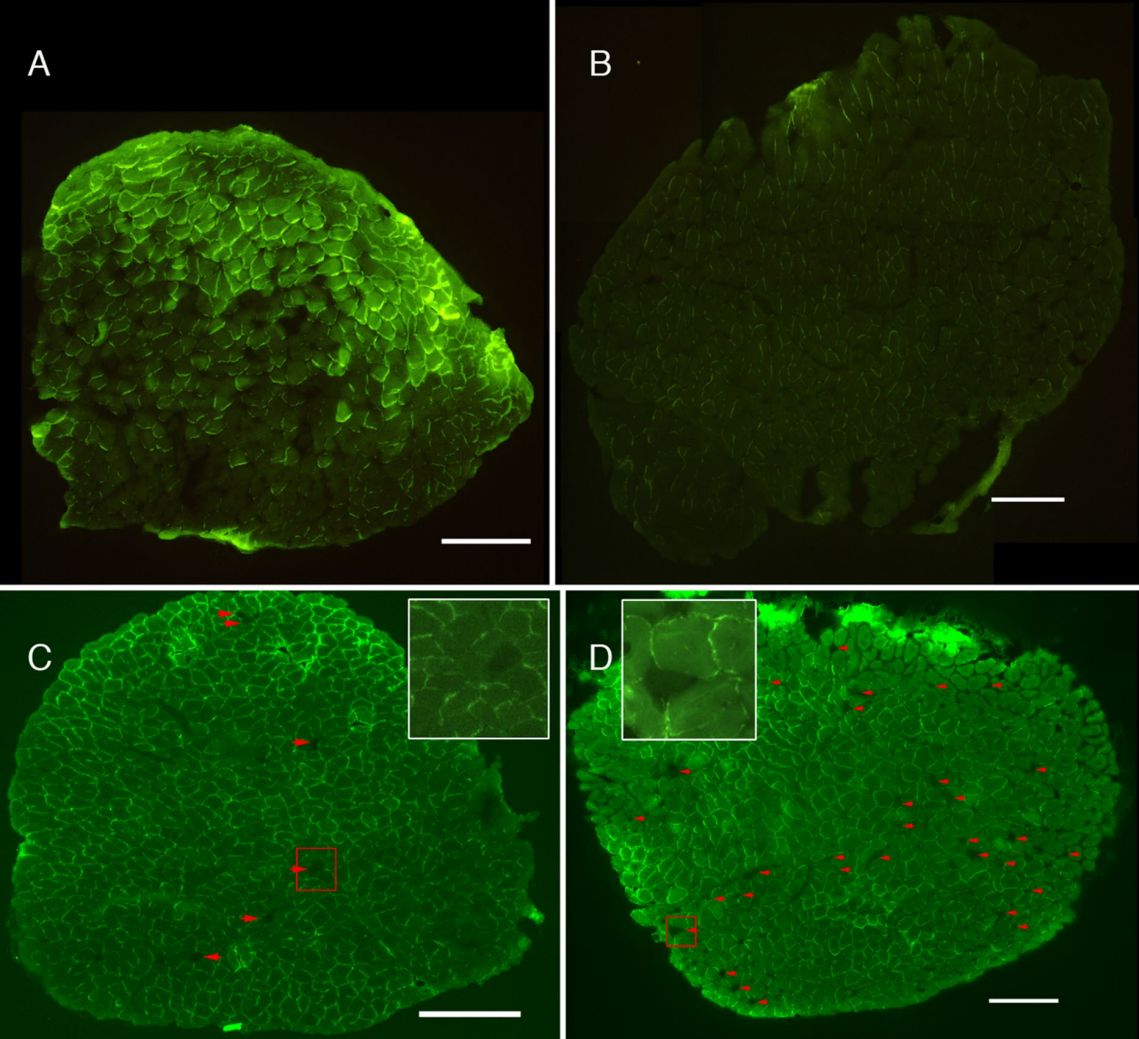
Age-related denervation of soleus muscle fibers visualized by the absence of 2-NBDG uptake. Validity of the 2-NBDG technique was confirmed by direct electrical stimulation of a soleus muscle’s posterior surface (upper edge in **A**). Fibers took up the label and were fluorescent near this surface while those located deeper away from the direct stimulation zone were not. Muscle fibers within the contralateral extensor digitorum longus were minimally fluorescent (**B**). In nerve-stimulated muscles, denervated muscle fibers were identified by low 2-NBDG fluorescence (**C** & **D**). Soleus muscles from younger mice (**C**) contained significantly fewer denervated muscle fibers (arrowheads) than older mice (**D**, data in Fig. 4). Insets in **C** & **D** are enlargements of red squares in companion images. Scale bars = 200 µm

Prior to force characterization mice were injected with an intravenous label (2-NBDG) which was subsequently taken into cells in proportion to their metabolic activity during stimulation. This technique was used to allow discrimination between recently activated and inactive fibers under the assumption that by nerve stimulation active muscle fibers should accumulate significant amounts of label while quiescent (denervated) fibers should not. This assumption was validated by a preliminary control experiment employing direct electrical stimulation of a soleus muscle’s posterior surface. When the muscle was sectioned, muscle fibers near the stimulation location at the posterior surface were strongly labeled (Fig. 2A) along with a small number of muscle fibers located deeper in the muscle (muscle surface stimulation also depolarizes underlying intramuscular nerve fibers which result in the activation of distant muscle fibers not directly stimulated by the surface electrode). Muscle fibers within the unstimulated regions of the muscle were minimally fluorescent and resembled muscle fibers from the unstimulated contralateral extensor digitorum longus muscle (negative control) (Fig. 2B).

**Fig. 3:**
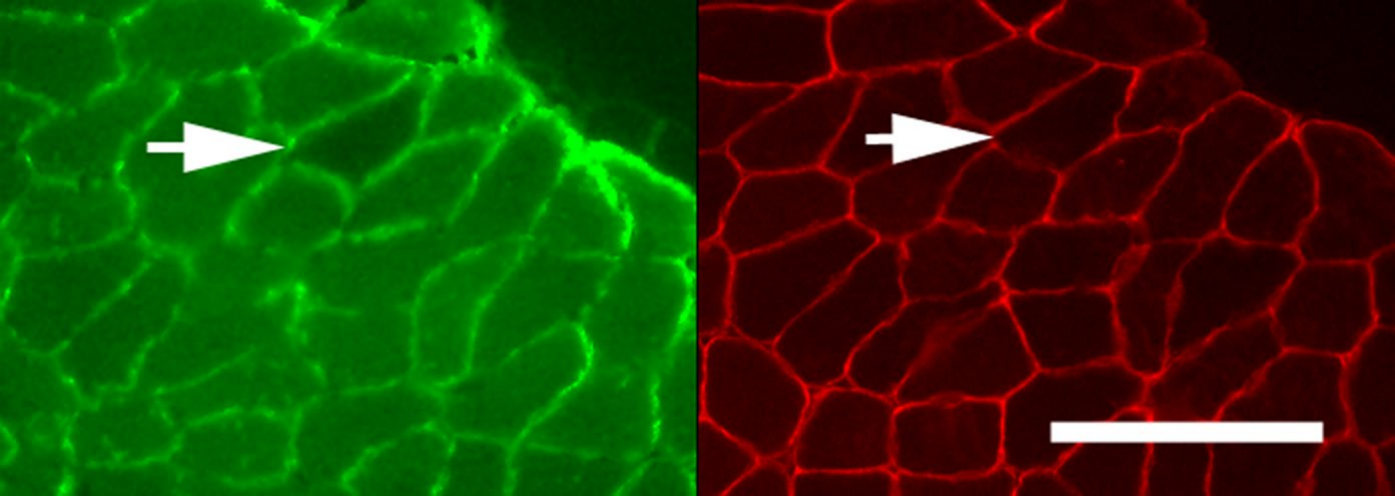
A presumptive denervated fiber having low 2-NBDG following nerve stimulation (arrow, left panel) is identified as a fiber rather than a hole or a gap in the section by having been immunostained for dystrophin (arrow in right panel). Scale bar = 100 µm

At the end of the force characterization protocol, the nerve of each muscle was continuously stimulated to drive 2-NBDG accumulation within innervated muscle fibers. Denervated muscle fibers were then identified in sections by virtue of low 2-NBDG fluorescence (Fig. 2C-D). To verify that these were muscle fibers and not section gaps, we also conducted dystrophin immunostaining and showed that 2-NBDG negative fibers were encircled by dystrophin (Fig. 3).

**Fig. 4:**
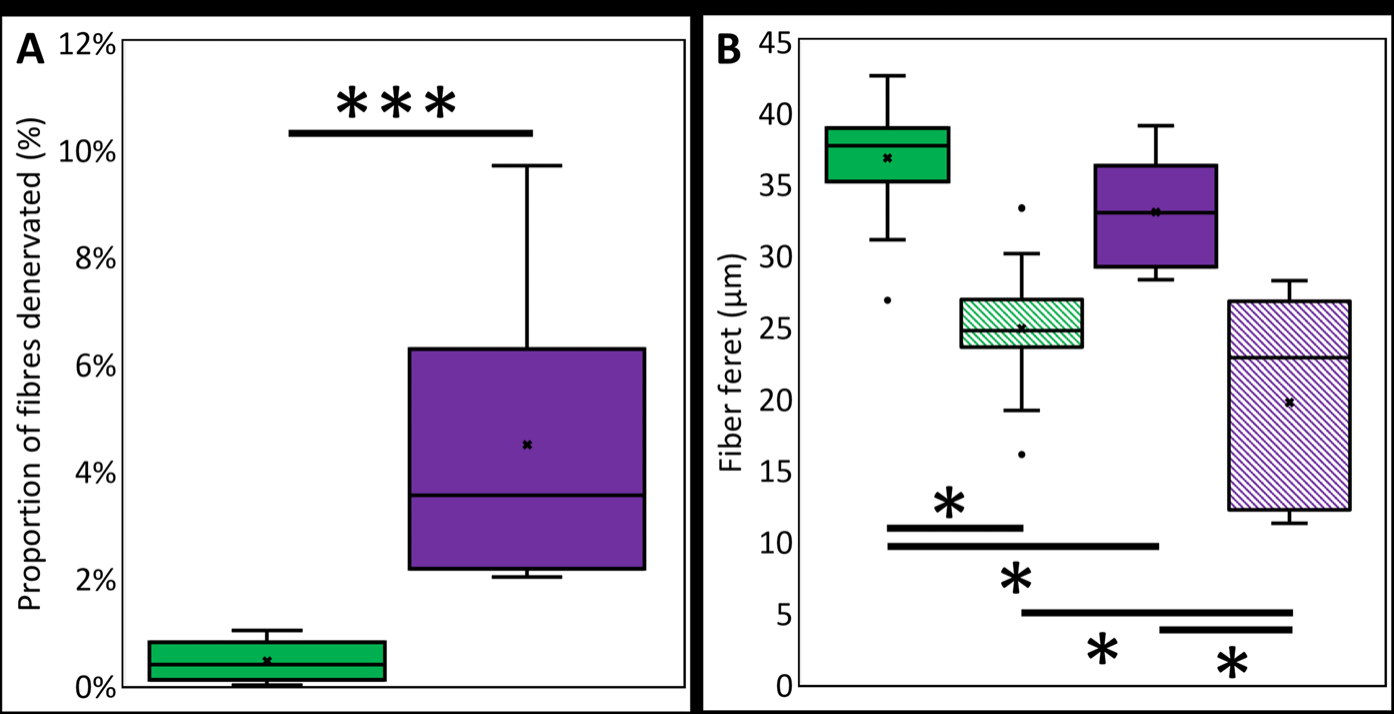
Denervated fiber prevalence and diameter in the soleus muscles of younger and older mice. The proportion of denervated fibers in muscles from younger (green) and older mice (purple) are shown in (**A)**. The diameter of innervated (solid color) and denervated fibers (diagonal lines) from muscles of younger and older mice are shown in (**B**). N = 13 for younger and 10 for older mice. *p < 0.050; ***p < 0.001 (color figure online)

Denervated fibers were found in soleus muscles from both young and older mice, but muscles from younger mice had significantly fewer denervated fibers (0.5 ± 0.4% vs. 4 ± 2%, p < 0.001, younger and older respectively) (Fig. 4A). The average diameter of denervated muscle fibers was smaller than innervated fibers in both younger (36.8 ± 3.8 µm innervated vs. 24.9 ± 4.3 µm denervated, p < 0.001) and older animals (33.0 ± 3.7 µm innervated vs. 19.7 ± 7.2 µm denervated, p < 0.001) (Fig. 4B). Denervated muscle fibers were smaller in older than in younger animals (24.9 ± 4.3 vs. 19.7 ± 7.2 µm, p = 0.016) (Fig. 4B). Moreover, the denervated muscle fibers of older animals exhibited greater size variability indicating that denervation was occurring progressively.

### Functional impact of denervated fibers

**Fig. 5:**
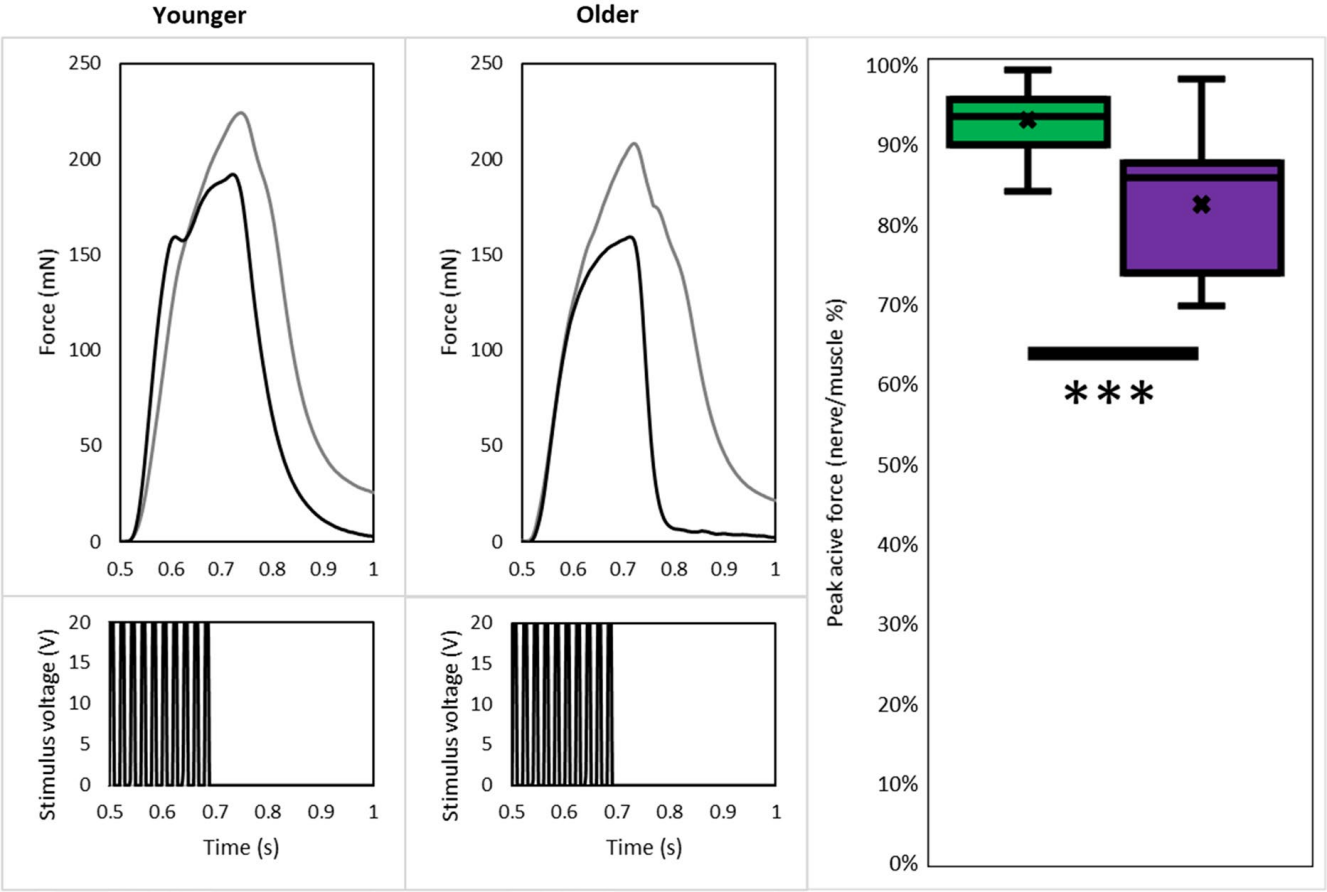
Age-related differences in peak active muscle tension from nerve compared to direct stimulation. To measure the functional impact of denervation, peak active tension via nerve stimulation was compared to peak active tension via direct stimulation of soleus muscles from younger and older mice. Representative traces are shown to the left (black: nerve, grey: muscle) while group results from younger (purple, n = 13) and older (purple, n = 10) muscles are shown on the right. There was a significant age-related decrease in peak active tension (nerve/muscle %). ***p < 0.001. Stimulus voltage indicates the pattern of stimulus rather than voltage as this was used to trigger an external stimulator (color figure online)

The functional impact of denervation was measured by comparing the peak force from nerve stimulation (denervated fibers would be unstimulated and remain inactive) to direct muscle stimulation (all fibers activated). When maximal force elicited from nerve stimulation was expressed as a percentage of that produced via muscle stimulation (nerve vs. muscle; NvM), we found that the presence of denervated fibers cost the young muscles 7% of their maximal force generating potential (NvM: 93 ± 4%), whilst denervated fibers in older muscles cost 18% of maximal force potential (NvM: 82 ± 9%). The functional consequence of denervation overall was more severe in older mice than younger (p < 0.001), (Table 1, Fig. 5). However, since we were able to quantify the number of denervated fibers in each muscle we were able to establish that each denervated fiber accounted for more of the NvM force deficit in younger compared to older muscles (− 2 ± 2% vs. − 0.7 ± 0.3%, p < 0.001) (Table 1).

**Table 1:** Age-related differences in muscle strength and contributions from muscle atrophy and denervation

	Younger	Older	Difference
Histological			
Total number of fibers	853 ± 153	905 ± 187	+ 6%
Number of denervated fibers	4 ± 3	36 ± 27	+ 900%***
Innervated fiber diameter (µm)	36.8 ± 3.8	33.0 ± 3.7	− 10%*
Denervated fiber diameter (µm)	24.9 ± 4.3	19.7 ± 7.2	− 21%*
Proportion denervated (%)	0.5 ± 0.4%	4 ± 3%	+ 785%*
CSA (mm^2^)	0.91 ± 0.18	0.84 ± 0.04	− 7%
CSA adj. (mm^2^)	0.9 ± 0.18	0.83 ± 0.04	− 8%
Functional			
Raw force from nerve stim. (mN)	206 ± 5	172 ± 10	− 17%***
Nerve force compared to TFGC (%)	93 ± 4%	82 ± 9%	− 12%***
Force deficit per denervated fiber (%)	− 2 ± 2%	− 0.7 ± 0.3%	− 135%**
Specific force (mN/mm^2^)	240 ± 42	200 ± 12	− 17%*
Adj. specific force (mN/mm^2^)	240 ± 41	202 ± 13	− 16%*

*CSA* total cross-sectional area occupied by muscle fibers; *Adj* adjusted (for the area occupied by denervated fibers); *stim* stimulated; *TFGC* total force generating capacity, measured by supramaximal stimulation of the muscle belly at its optimal length; *p < 0.050; **p < 0.010; ***p < 0.001

When peak force was corrected for age-related differences in total cross-sectional area, muscles from older mice still produced less specific force than muscles from younger mice (240 ± 42 vs. 200 ± 12mN/mm^2^, p = 0.040) (Table 1). These figures were calculated from total cross-sectional area which includes the area occupied by denervated muscle fibers. Since denervated fibers remain quiescent during nerve stimulation total cross-sectional area underestimates specific force. However, total cross-sectional area adjusted for denervated fibers did not significantly alter specific-force values (240 ± 41 vs. 202 ± 17mN/mm^2^, p = 0.049) (Table 1). These findings showed that age-related declines in muscle force are only partly explained by differences in muscle cross-sectional area (including adjustment for the area occupied by denervated muscle fibers).

## Discussion

This investigation documented the contribution of muscle atrophy and denervation to age-related decline in muscle active tension using a novel technique to identify denervated muscle fibers. We did this by reconciling data on muscle function and histology from muscles exposed to a label (2-NBDG) which accumulated within recently activated muscle fibers. We found a 17% age-related deficit in muscle force (240 ± 42 vs. 200 ± 12mN/mm^2^) (Table 1), 10% decline in fiber diameter (36.8 vs 33.0 µm young vs old, Fig. 4B) and almost eightfold increase in denervated fiber prevalence (0.5 ± 0.4% vs. 4 ± 2%, p < 0.001, younger and older respectively) (Fig. 4A) (Table 1). However, the 10% reduction in fiber size was insufficient to account for the 17% decline in muscle strength when muscle force was normalized to total fiber cross-sectional area. By excluding denervated fibers from cross-sectional area, we found a 16% age-related decline in muscle specific force. Our results show that loss of fiber size and denervation accounted for 6% of the decline in muscle active tension, while the remaining decline was not explained by these factors.

The identification of denervated muscle fibers has previously been conducted using three different methods: [1] visualization of lack of association between pre- and post-synaptic apparatuses at the neuromuscular junction; [2] NCAM/NaV_1.5_ fiber immunostaining; and [3] glycogen depletion. Investigators that have described denervation via the association between pre- and post-synaptic apparatuses have viewed features of the motor endplate by longitudinal or transverse sectioning or wholemount immunostaining (Chai et al. 2011; Gillon and Sheard 2015; Valdez et al. 2010). Motor endplates are identified by the presence of a post synaptic fluorescent marker (typically fluorophore-conjugated α bungarotoxin), and endplates where fluorescent markers for the pre-synaptic apparatus (typically marked by immunolabeling of a synaptic vesicle protein) co-localize with post synaptic markers are considered innervated. By contrast, denervated motor end plates lack indicators for the presynaptic apparatus and endplates in various stages of deterioration are often fragmented and partially innervated (Valdez et al. 2012). This methodology typically establishes the innervation status of only a subset of fibers. Moreover, the functional status of endplates with a fragmented or partially innervated morphology is not established with such histological techniques, so the functional significance of partially innervated junctions is not well known (Slater 2020).

Data from developmental studies have demonstrated that immature pre-innervated muscle fibers express neural cell adhesion molecule (NCAM) on their sarcolemma (Sanes et al. 1986; Walsh and Doherty 1991). This observation has been used to identify denervated muscle fibers in both human and rodent samples (Gillon and Sheard 2015; Soendenbroe et al. 2019; Sonjak et al. 2019) and permit recalculation of specific muscle force production (Urbanchek et al. 2001) showing that muscle fiber denervation only partially accounts for age-related muscle force deficits. However, the use of NCAM immunohistochemistry as a reliable indicator of fiber denervation has been called into question as it has been found that experimental denervation elicits a blunted NCAM expression response amongst muscles of older mice (Gillon and Sheard 2015) potentially resulting in underestimation of the number of denervated muscle fibers. Uncertainty regarding the reliability of NCAM as a marker of denervated human fibers has been expressed (Soendenbroe et al. 2019). A more specific approach to identifying denervated muscle fibers utilizes the expression of NaV_1.5_ (a tetrodotoxin-insensitive voltage gated sodium channel). NaV_1.5_ is not normally expressed in adult skeletal muscles but its expression has been observed in the muscle fibers of older rats (Rowan et al. 2012) and within experimentally denervated mouse muscles (Gillon and Sheard 2015). A feature of these histological approaches is that they offer indirect visualization of denervated muscle fibers with no indication of the functional status of any deteriorating nerve connections. To circumvent this problem other groups have used a functional approach by continuously electrically stimulating the sciatic nerve so that innervated fibers consume their glycogen stores (Ballak et al. 2014a), thereby marking functionally denervated fibers by virtue of their retention of glycogen which can later be detected histologically. Whilst this method has been used successfully in a predominantly fast twitch muscle (plantaris), glycogen depletion is problematic in many mouse muscles since background glycogen levels are too low to permit introduction of a depletion-based contrast (our unpublished observations).

Given the limitations of the previous techniques for identifying all functionally denervated fibers within a muscle, we opted to leverage contraction-responsive glucose influx to fluorescently label functionally innervated fibers. We employed activity-dependent uptake of a non-metabolized fluorescent deoxyglucose analog (2-NBDG). A version of this technique (using radioactive deoxyglucose) has previously been used to visualize the fibers of individual motor units (Toop et al. 1982) but not to identify denervated fibers; therefore our implementation is a novel application. Our observations of age-related differences in the prevalence of denervated fibers are concordant with those of other investigators (Ballak et al. 2014a). There were no discernable patterns in the location or grouping of denervated fibers, suggesting that there were no regional factors within the muscle contributing to its pathogenesis and that it was unlikely that the denervated fibers had been members of the same motor unit. Denervated fibers were smaller than innervated fibers in the muscles of both younger and older mice, consistent with the progression of denervation atrophy as documented in studies on rodent and human muscle (Rowan et al. 2011; Rowan et al. 2012; Soendenbroe et al. 2019, Sonjak et al. 2019). Variation in denervated fiber diameter in the muscles of older mice was larger than in the muscles of younger mice, suggesting that denervation was an ongoing process that was occurring cumulatively and progressively. Regardless of etiology, fibers that become denervated in the muscles of younger subjects can be reinnervated by collateral sprouts from neighboring nerve terminals (motor unit expansion) as seen in mice and humans (Sonjak et al. 2019; Valdez et al. 2012). Reinnervation of denervated fibers by motor unit expansion appears less likely to occur in the muscles of older individuals (Piasecki et al. 2018; Skinner et al. 2021), leading to longer periods of denervation and fiber atrophy (Purves-Smith et al. 2012; Rowan et al. 2011, 2012; Skinner et al. 2021).

Our study provides a direct assay of functional denervation with ageing across the entire muscle and shows that some fibers are functionally denervated from an early age, and that the proportion of such fibers increases with the passage of time. What the assay does not show is the process of reinnervation that is thought to follow denervation at many junctions (Edstrom et al. 2007; Larsson 1995; Sonjak et al. 2019). Therefore, it is possible that the relatively small number of denervated fibers in the young animals may have later become reinnervated. It is possible to use current and existing data to estimate the significance of denervation and reinnervation. Mouse soleus muscles have approximately 850–900 fibers comprising approximately 20 motor units (Taxt 1983) so motor unit size is, on average, 40–45 fibers. Death of motor neurons in normal ageing would result in denervation of all fibers innervated at the time of the neuron’s death, and since about 25% of neurons are lost from the mouse lumbar spinal cord in normal ageing (Gillon et al. 2018), we expect that five neurons should be lost from the soleus motor pool and this process would result in the denervation of approximately 200 fibers across the mouse lifespan. Our observations show that an average of only one fifth of that number of fibers were functionally denervated, suggesting that four fifths of the fibers that are denervated by the age-related loss of motoneurons subsequently become reinnervated by sprouts from neighboring nerve terminals, a process that results in progressive increase in the size of surviving motor units (Valdez et al. 2012). These observations further support the notion that cycles of denervation and reinnervation are a feature of normal neuromuscular ageing, and that long-term denervation of fibers is relatively uncommon. More recent studies examining denervation and reinnervation cycles in rodents and humans reach similar conclusions (Sonjak et al. 2019; Skinner et al. 2021). The reduced diameter of denervated fibers (Fig. 4B) suggests that reinnervation does not typically occur immediately, and the presence of very small diameter denervated fibers in the old muscles is consistent with a subset of fibers failing to become reinnervated and subsequently undergoing significant disuse atrophy.

Muscle atrophy is an obvious likely contributor to muscular weakness in old age, and atrophy is typically identified by decline in muscle mass and cross-sectional area. In this study we documented a small but significant decline in average fiber diameter amongst mouse soleus fibers, but even after accounting for differences in denervated fiber prevalence and the cross-sectional area they contribute, a force discrepancy remains (Table 1). The area contribution of denervated fibers did not match their functional impact (according to nerve/muscle stimulation, Table 1). These findings were similar to those of other investigators who also measured the functional impact of denervation (Urbanchek et al. 2001; Ham et al. 2020). Fiber denervation may further impact on weakness by reducing efficiency of force transmission between active fibers and tendons. Since force is transmitted both longitudinally (fiber to tendon) and laterally (fiber to circumferential connective tissue to neighboring fibers), force generated by active fibers can be transmitted to tendons partly via neighboring fibers. Active fibers adjacent to compliant inactive fibers are likely to contribute some of their force to the passive lengthening of their neighbors (Sheard 2000), thereby reducing the efficiency of force delivery and increasing the functional impact of inactive fibers beyond the level expected by their area.

Besides changes in muscle structure, muscle atrophy, fiber denervation, and reduced force transmission, single fiber weakness may also contribute to the functional decline. One factor that can influence force generated by single fibers (accommodating differences in fiber diameter) is fiber type transformation, though there is relatively little change in mouse soleus fiber type composition with age (Hill et al. 2020) indicating that this was not a major contributor to muscle weakness. Age-related changes in the arrangement and density of sarcomeres within fibers could also influence the force generating capacity of single fibers, without necessarily changing fiber diameter. The force generating capacity of individual muscle fibers from younger and older mice has been characterized by other investigators (Hvid et al. 2011; Li and Larsson 1996; Reid et al. 2012). When aggregated, the results of the single fiber studies show no age-related changes in the specific force produced by muscle fibers.

Muscles from older mice were found to produce less active tension and more passive tension at L_0_ than younger mice, results that corroborate previous observations (Brown et al. 1999; Rosant et al. 2007; Winegard et al. 1996). Maximum muscle tension in this investigation was controlled for muscle length, stimulus voltage and duration. However, differences were observed between the force-frequency curves of muscles from younger and older mice. Therefore, to ensure maximal muscle force was recorded, muscles were stimulated at individually controlled frequencies. The muscles of older mice were were less responsive to changes in stimulation frequency and produced half maximal force at a frequency 4 Hz greater than muscles from younger mice. The age-related decline in stimulation frequency response may be attributed to dysregulation of calcium flux (Ha et al. 1999) or calcium leak. Possible sources of calcium leak include the sarcoplasmic reticulum or the muscle fiber membrane, both of which have been implicated in muscle aging (Andersson et al. 2011; Ramaswamy et al. 2011). Compromised sarcolemma integrity has previously been observed near the myotendinous junctions (MTJs) of muscle fibers from older mice (Lal and Sheard 2015). Membrane tears localized to MTJs might be involved in the pathogenesis of MTJ and tendinous elongation (Lal and Sheard 2015; Nielsen et al. 2018; Lal et al. 2021) and they may also lead to the activation of calcium dependent proteases which degrade sarcomeres (Tidball et al. 1995). Age-related proliferation of connective tissue including lengthening of both tendon and MTJ along with fiber shortening via loss of sarcomeres (Hooper 1981) are likely contributors to the observed functional changes including the increase in passive tension at L_0_.

## Conclusion

The application of a new method using activity dependent uptake of a fluorescent marker shows that muscle fibers with no functional nerve contact are present throughout adulthood in the mouse soleus, and that the proportion of such fibers increases with age. Old muscles generate less force than young muscles, but this deficit is only partly attributable to denervation. Fibers in old muscles are smaller than in young, and whilst this also contributes to the age-related loss of force there is a further remaining deficit that potentially arises as a consequence of age-related changes in sarcomeric proteins, fiber length, and connective tissue remodeling. The specific contributions of these and any other factors contributing to loss of force-generating capacity are yet to be determined.
